# Kir6.2-D323 and SUR2A-Q1336: an intersubunit interaction pairing for allosteric information transfer in the K_ATP_ channel complex

**DOI:** 10.1042/BCJ20190753

**Published:** 2020-02-11

**Authors:** Sean Brennan, Hussein N. Rubaiy, Saba Imanzadeh, Ruth Reid, David Lodwick, Robert I. Norman, Richard D. Rainbow

**Affiliations:** 1 Department of Molecular and Clinical Pharmacology, Institute of Translational Medicine, University of Liverpool, Sherrington Building, Ashton Street, Liverpool, Merseyside L69 3GE, U.K.; 2 Department of Cardiovascular Sciences, University of Leicester, Clinical Sciences Wing, Glenfield General Hospital, Leicester LE3 9QP, U.K.; 3 Department of Molecular and Cell Biology, University of Leicester, Henry Wellcome Building, Lancaster Road, Leicester LE1 7RH, U.K.; 4 Centre for Biological Engineering, Loughborough University, Loughborough LE11 3TU, U.K.; 5 Leicester Medical School, University of Leicester, George Davis Centre, Lancaster Road, Leicester LE1 7RH, U.K.

**Keywords:** ABC transport proteins, KATP, molecular interactions, potassium channels

## Abstract

ATP-sensitive potassium (K_ATP_) channels are widely expressed and play key roles in many tissues by coupling metabolic state to membrane excitability. The SUR subunits confer drug and enhanced nucleotide sensitivity to the pore-forming Kir6 subunit, and so information transfer between the subunits must occur. In our previous study, we identified an electrostatic interaction between Kir6 and SUR2 subunits that was key for allosteric information transfer between the regulatory and pore-forming subunit. In this study, we demonstrate a second putative interaction between Kir6.2-D323 and SUR2A-Q1336 using patch clamp electrophysiological recording, where charge swap mutation of the residues on either side of the potential interaction compromise normal channel function. The Kir6.2-D323K mutation gave rise to a constitutively active, glibenclamide and ATP-insensitive K_ATP_ complex, further confirming the importance of information transfer between the Kir6 and SUR2 subunits. Sensitivity to modulators was restored when Kir6.2-D323K was co-expressed with a reciprocal charge swap mutant, SUR-Q1336E. Importantly, equivalent interactions have been identified in both Kir6.1 and Kir6.2 suggesting this is a second important interaction between Kir6 and the proximal C terminus of SUR.

## Introduction

The ATP-sensitive potassium (K_ATP_) channel forms a sub-group of the inward-rectifying potassium ion channel family that are voltage-insensitive and ubiquitously expressed. The key roles for K_ATP_ channels include energy-sparing in cardiac and skeletal muscle [1,2], neuroprotection [3,4], regulation of insulin secretion from pancreatic β-cells [5], and regulation of vascular tone [6]. K_ATP_ channels were once front-line targets used in the treatment for type-II diabetes [5], hypertension or angina [6–8], indicating that they represent clinically important and ‘druggable’ proteins.

K_ATP_ channels are unique amongst the inwardly rectifying K^+^ channel family due to the requirement of a large accessory protein for its surface expression. The channel is a complex formed from a Kir6 tetramer co-assembled with a tetramer of sulphonylurea receptor (SUR) proteins that form a heteroctameric functional complex [9,10]. Kir6 subunits have intracellular N- and C-termini and two transmembrane segments M1 and M2, separated by a P-loop that forms the outer mouth of the weakly rectifying K^+^ selective pore [5]. The SUR subunits have extracellular N- and intracellular C-termini and 17 transmembrane segments.

Due to a C-terminal endoplasmic reticulum retention sequence (RKR), the Kir6 cannot express at the membrane surface without the SUR interaction, which presumably masks this motif. There are two mammalian Kir6 family members, Kir6.1 and Kir6.2. The Kir6.2 pore-forming subunit has an ATP binding site on the C terminus that inhibits the current through the weakly rectifying K^+^ selective pore [11]. Kir6.2 can functionally express at the cell surface if the subunit is truncated at the C-terminal 26 or 36 residues (Kir6.2ΔC26, Kir6.2ΔC36) and shows current that is ATP sensitive, albeit with a 10-fold right-shifted IC_50_ of ∼120 µM [12]. Similar Kir6.1 truncations still allow the protein to traffic to the membrane, however, the recorded current is very small, not metabolically sensitive and can only be inhibited by the Kir6.1 pore blocker, PNU37883A (Kir6.1ΔC48 and Kir6.1ΔN13/ΔC48) [13].

Co-assembly of Kir6.2 with an SUR enhances the ATP sensitivity of the complex, with reports in the literature showing an ATP-sensitivity in Kir6.2/SUR2A (the cardiac and skeletal muscle isoform) of ∼20 µM [14–17]. In addition to the enhanced ATP sensitivity, the SUR imparts ADP and nucleotide diphosphate (NDP) sensitivity to the current [18–20]. Furthermore, the accessory subunit imparts sulphonylurea drug sensitivity to the channel which includes activators, such as pinacidil [14,21], and inhibitors, such as glibenclamide [14,22,23]. This, and given the ubiquity of expression, led to an interest in K_ATP_ as a pharmacologically useful therapeutic target that continues to this day.

The Kir6.1 containing channel complex shows markedly different properties to the Kir6.2. This isoform, most often associated with the vasculature, is reported to be relatively ATP-insensitive and dependent on NDP's for its activity [13,24]. The Kir6.1/SUR2B channel complex is also highly regulated by protein kinase activity in the vasculature, which is attributed to phosphorylation of either the SUR or Kir6 component of the channel complex [25–27]. The fact that not all modulators of the full complex act via the pore-forming subunit led to the hypothesis that there must be regions of interaction between the pore forming and β-subunits that allow the exchange of information between the two subunits, i.e. binding of a drug to the SUR causing an allosteric modification to the gating of the Kir6 pore.

**Figure 1.**
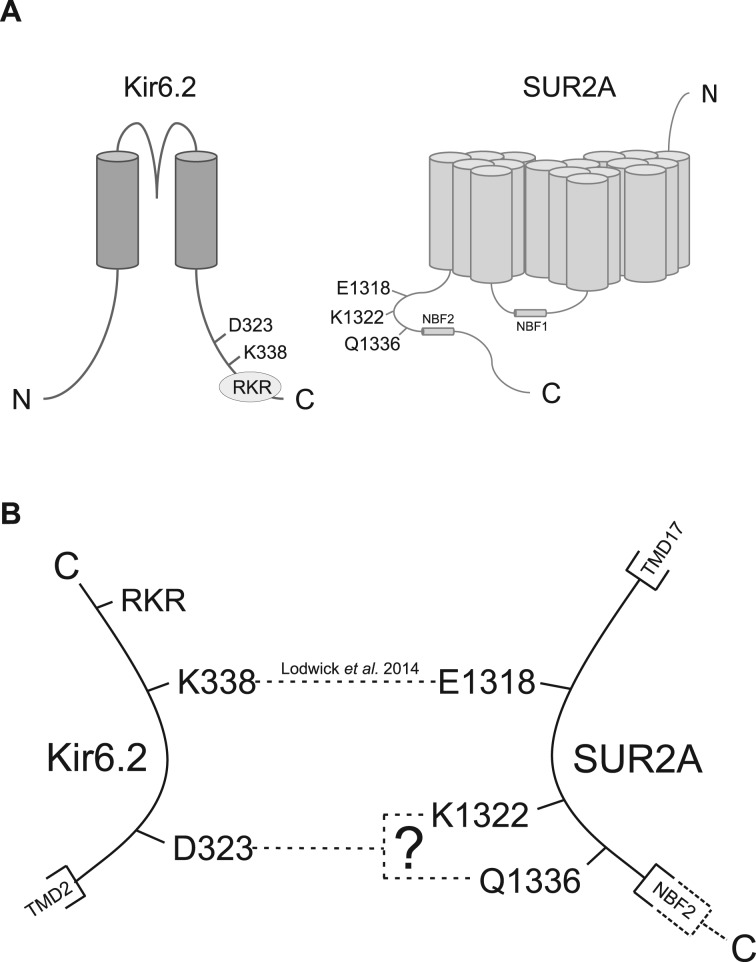
Putative salt bridges between the proximal C terminus of SUR2A and the C terminus of Kir6.1. (**A**) Cartoon showing the residues thought to be key in transferring gating information between SUR2A and Kir6.2 subunits. (**B**) Schematic showing the interaction between SUR2A-E1318 and Kir6.2-K338 identified in our previous study [14] and the putative interaction between Kir6.2-D323 and SUR2A-K1322 or Q1336.

Our first publication in this area identified a 64-amino acid sequence, between residues 1294 and 1358 on the SUR2 subunit, that was a key region of interaction between the Kir6 and SUR2 family, as identified in co-immunoprecipitation experiments [15]. When co-expressed with full length Kir6.2/SUR2A subunits, this small protein fragment was able to act like a dominant negative on channel function by disrupting functional expression [15,28]. Our subsequent manuscript demonstrated that within this region of SUR2 there was a minimally interacting fragment of the C terminus (1318–1337), and a corresponding region of interaction within the C-terminal 75 residues of Kir6.2-(316–390) [14]. Two putative residues of interaction on the Kir6.2 C terminus were identified (D323 and K338), and 3 on the SUR C-terminal region (E1318, K1322 and Q1336) (Figure 1). Co-immunoprecipitation experiments, and electrophysiological studies, demonstrated that the K338 and E1318 residues were able to interact, demonstrated by performing charge swap experiments where a Kir6.2-K338E mutation, when co-expressed with SUR2A-E1318R, fully restored function to the channel, including pinacidil, glibenclamide, ATP and ADP sensitivity [14]. Corresponding charge swap mutations in Kir6.1 (R347E), also fully restored pinacidil and glibenclamide sensitivity. These data demonstrated that the Kir6.2-K338 (Kir6.1-R374) formed a salt bridge with the SUR2A-E1318 residue to allow the transfer of information between the subunits. Disruption of this salt bridge with a mutation in either the pore-forming subunit, or the accessory subunit, disrupted the normal channel function [14]. The recent publication of cryo-electron microscopy structures for the Kir6.2/SUR1 complex have given us new information regarding the structure and putative interactions between the subunits [29–32].

Co-immunoprecipitation experiments in our previous study demonstrated that there was a second potential salt bridge between Kir6.2-D323 and either SUR2A-K1332 or Q1336 [14]. In this study, we used electrophysiological measurements of membrane currents to test the hypothesis that there was a salt bridge between putative interacting residues. To investigate this, a charge swap at residue Kir6.2-D323 was used to disrupt the putative interaction, and the salt bridge ‘restored' by co-expressing a reciprocal charge swap on the SUR2A-K1332 or -Q1336 to investigate the transmission of regulatory information between subunits.

## Methods

### Cell culture

Human embryonic kidney (HEK)-293 cells were transiently transfected with both Kir6.2 and SUR2A subunits, and mutant subunits as indicated, 48 h before patch-clamp investigations. Cells were released from the plate by a 2 min trypsin digestion (0.5 g/l trypsin, 0.2 g/l EDTA; Sigma), followed by repeated washing with sterile 2 mM Ca^2+^ Tyrode's solution (2CaT) (5 mM KCl, 135 mM NaCl, 0.33 mM NaH_2_PO_4_, 5 mM sodium pyruvate, 5 mM glucose, 10 mM HEPES, 2 mM CaCl_2_ and 1 mM MgCl_2_), adjusted to pH 7.4 with NaOH. Cells were maintained in 2CaT at room temperature until use. For excised inside-out patch recording, cells were cultured and transfected on coverslips 48 h before use. Patches were excised from the cells already adhered to the coverslip.

### Molecular biology

Point mutants were produced by overlap PCR with inserts confirmed by DNA sequencing. For electrophysiology, Kir6.0 wild-type (WT) and mutants were expressed in pcDNA3.1/myc/hisA (Invitrogen) and SUR2A WT and mutants expressed in pIRES2-EGFP-F [14,15].

### Patch clamp

For conventional whole-cell recording from HEK293 cells, electrodes were pulled from thick-walled filamented borosilicate glass to a resistance of 3–6 MΩ. Cells were continuously perfused at 32 ± 2°C at a rate of 5 ml min^−1^ with 2CaT solution. Pinacidil and glibenclamide were added as indicated in each figure. Currents were recoded using an Axopatch 200B amplifier, digitised using a Digidata 1440 interface and recorded to computer using pCLAMP10.7. Transfected cells were identified by EGFP-F fluorescence at 488 nm under mercury lamp illumination. Cells were voltage-clamped at 0 mV (approximate E_K_ for these solutions would be ∼−89 mV and so a large outward current was recorded). Membrane potentials were recorded for each cell by switching to current clamp prior to adding pinacidil. For excised inside-out patch recording, experiments were carried out at room temperature with the excised patch placed directly into the perfusion flow [14,15]. Pipette and perfusing solutions both contained 140 mM K^+^ so the pipette potential was held at 0 mV and currents recorded with a hyperpolarising step to −80 mV to avoid rectification seen with outward K_ATP_ currents.

### Data analysis

All patch clamp data was recorded and analysed in pCLAMP10.7, and further analysed in Microsoft Excel 2016. Figures were prepared, and statistical analysis was carried out, in Graphpad Prism 7. Data is presented in bar charts as mean ± S.D. with the individual data points superimposed; n is reported as a number of cells.

### Modelling putative interactions

Figures 11(C–G) and 12(A–C) were created using Pymol software. The cryo-EM structure of human pancreatic K_ATP_ channel (Kir6.2/SUR1) in the propeller conformation (PDB code:6C3P [32]) has been used for Figure 12.

The distance between D323 and R248 was measured using the measurement tool in Pymol. This measurement was done before (Figure 12B) and after modelling the D323K point mutation (Figure 12C). Virtual D323K point mutation was carried out using the mutagenesis tool in Pymol. In this mutagenesis, the most common rotamer of Lysine was chosen, indicated as a percentage associated with each potential conformation of Lysine in the protein, calculated by Pymol. These percentage values are based on the commonality of all the possible conformations of residues in proteins.

## Results

### Kir6.2-D323K co-expressed with WT-SUR2A results in a constitutively active current

**Figure 2.**
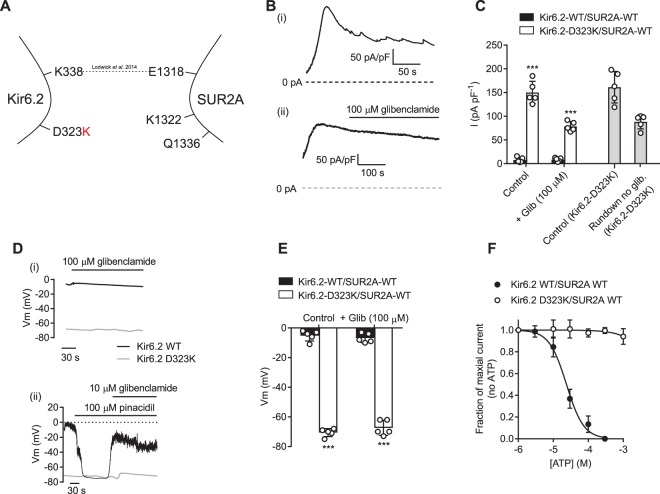
Kir6.2-D323K mutation yields a constitutively active Kir6.2 subunit that is insensitive to sulphonylureas and ATP. (**A**) Cartoon showing the Kir6.2-D323K mutation, disrupting the putative salt bridge to SUR2A. (**B**) Whole cell current recording held at 0 mV, from HEK293 cells transiently transfected with Kir6.2-D323K/SUR2A-WT, showing constitutive activity that runs down over time (i) and that is insensitive to 100 µM glibenclamide. (**C**) Mean data from HEK293 cells transiently transfected with Kir6.2-D323K/SUR2A-WT showing a substantial K^+^ current in control conditions compared with K_ATP_-WT (Kir6.2/SUR2A) (*** *P* < 0.0001, Two-Way ANOVA with Holm-Sidak's post test), where there was no difference between glibenclamide treatment and run down of the current (*n* = >5 for each group). (**D**) (i) membrane potential (Vm) recordings in K_ATP_-WT and Kir6.2-D323K/SUR2A-WT transiently transfected HEK293 cells. (ii) membrane potential recordings in both cell types showing pinacidil-induced hyperpolarisation and glibenclamide-induced depolarisation in K_ATP_-WT transfected HEK293 cells. There was no effect on the membrane potential of Kir6.2-D323K/SUR2A-WT transfected cells. (**E**) Mean data showing the membrane potential in control conditions and in 100 µM glibenclamide in K_ATP_-WT and Kir6.2-D323K/SUR2A-WT transfected cells. There was no significant difference in the effect of glibenclamide on the membrane potential of K_ATP_-WT or Kir6.2-D323K/SUR2A-WT, however the Kir6.2-D323K mutant caused a significantly more hyperpolarised membrane potential in control and glibenclamide conditions (*** *P* < 0.0001, Two-Way ANOVA with Holm-Sidak's post test, *n* > 5 for each group). (**F**) ATP concentration response data recorded from excised inside-out patches. K_ATP_-WT patches had an IC_50_ for ATP of 23.8 ± 1.2 µM, but there was no effect of ATP in the Kir6.2-D323K/SUR2A-WT mutant (*n* = 6 for each subunit combination).

In our previous study, the point mutation Kir6.2-K338E caused a 10-fold leftwards shift in the EC_50_ for pinacidil, a 100-fold rightwards shift in the IC_50_ for glibenclamide and small rightwards shift in ATP sensitivity when co-expressed with WT SUR2A [14]. The Kir6.2-D323K mutation was, however, somewhat more severe in terms of its phenotype when co-expressed with WT SUR2A, showing significant constitutive activity (Figure 2B,C) that was unresponsive to glibenclamide in whole-cell recording (Figure 2B,C), and unresponsive to ATP or ADP in excised inside-out patch recording (Figure 2F). The confirmation of a functional potassium current expression was demonstrated by measuring the resting membrane potential of the cells co-expressing Kir6.2-D323K/SUR2A-WT and showing a mean resting membrane potential of −73.4 ± 4.1 mV (*n* = 6) (Figure 2D,E).

**Figure 3.**
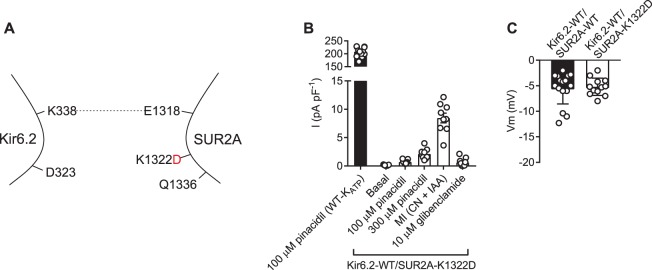
Kir6.2-WT co-expressed with SUR2A-K1322D forms a channel that functionally expresses little current. (**A**) Cartoon showing the SUR2A-K1322D mutation, disrupting the putative salt bridge to Kir6.2. (**B**) Mean whole cell current recording held at 0 mV, from HEK293 cells transiently transfected with Kir6.2-WT/SUR2A-K1322D, showing some sensitivity to pinacidil, metabolic inhibition (with cyanide and iodoacetic acid) and to 10 µM glibenclamide (*n* = 9). The current produced was too small to record accurate pinacidil, glibenclamide or ATP sensitivity. (**C**) Mean membrane potential for both K_ATP_-WT and Kir6.2-WT/SUR2A-K1322D was not significantly different (*t*-test, *n* > 12 for each group), indicative of negligible hyperpolarizing current.

Biochemical evidence [14] indicates that the Kir6.2-D323K mutation could co-immunoprecipitate with either the SUR2A-K1322D or -Q1336E charge swaps, so suggesting an interaction. It was therefore hypothesised that expression of the SUR2A mutants with a WT Kir6.2 would also impair normal current modulation by sulphonylurea drugs and ATP. Co-expression of Kir6.2-WT and SUR2A-K1322D resulted in a very limited current that was not constitutively active, was still modulated by sulphonylureas and could be potentiated by metabolic inhibition of the HEK293 cells (2 mM cyanide and 1 mM iodoacetic acid) (Figure 3B). The resting membrane potential of the Kir6.2-WT/SUR2A-K1322D expressing cells was not different from that of K_ATP_-WT (Figure 3C). These data show that the Kir6.2-WT/SUR2A-K1322D complex is able to form functional channels, however there was sufficient disruption of the channel that function was very limited.

**Figure 4.**
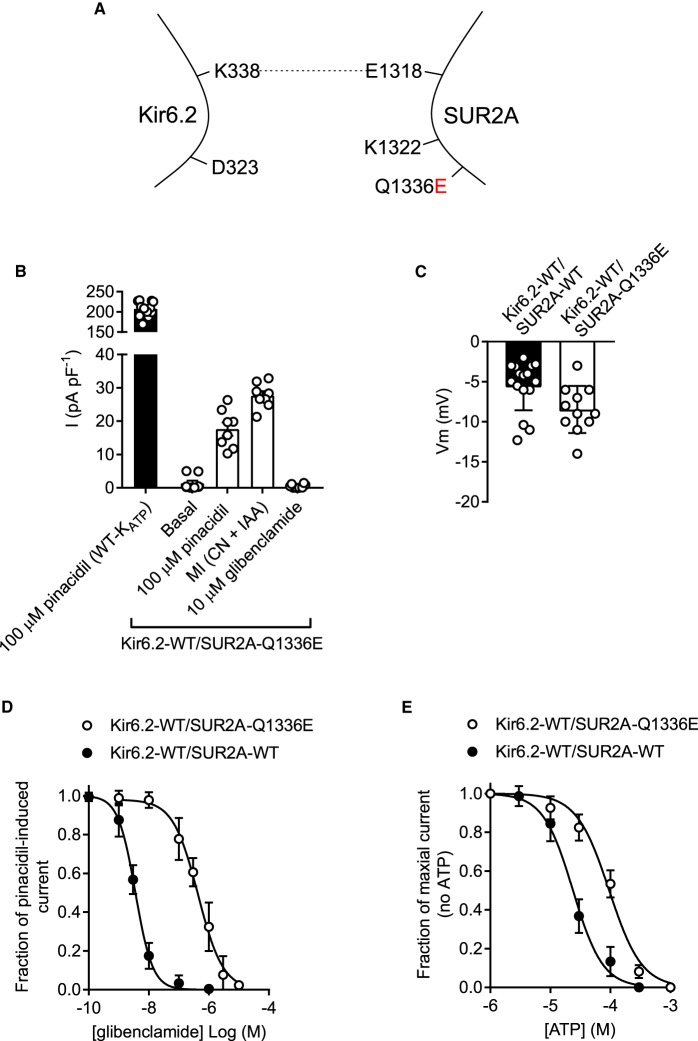
Kir6.2-WT co-expressed with SUR2A-Q1336E forms a channel that functionally expresses but has reduced sensitivity to glibenclamide and ATP. (**A**) Cartoon showing the SUR2A-Q1336E mutation, disrupting the putative salt bridge to Kir6.2. (**B**) Mean whole-cell current recording held at 0 mV, from HEK293 cells transiently transfected with Kir6.2-WT/SUR2A-Q1336E, showing sensitivity to pinacidil, metabolic inhibition (with cyanide and iodoacetic acid) and to 10 µM glibenclamide (*n* = 8). The current produced was small but was robust enough to record glibenclamide and ATP sensitivity. (**C**) Mean membrane potential for both K_ATP_-WT and Kir6.2-WT/SUR2A-Q1336E was not significantly different (*t*-test, *n* > 12 for each group). (**D**) Glibenclamide concentration-inhibition data recorded from WT-K_ATP_ and Kir6.2-WT/SUR2A-Q1336E expressed in HEK293 cells. Inhibition of the Kir6.2-WT/SUR2A-Q1336E complex by glibenclamide was significantly right shifted (IC_50_ of 4.3 nM to 433 nM, *P* < 0.002, *t*-test *n* = 6 for each group). (**E**) ATP-sensitivity was also right shifted from an IC_50_ in control excised inside-out patches of 23.4 µM to 90 µM in the Kir6.2-WT/SUR2A-Q1336E channel complex (*P* < 0.0001, *t*-test, *n* = 6 for each group).

Co-expression of Kir6.2-WT with the SUR2A-Q1336E mutant yielded a more robust current density in the HEK293 cells (Figure 4B) with no significant change in basal constitutive current or resting membrane potential in cells compared with those expressing K_ATP_-WT (Figure 4B,C). Kir6.2-WT/SUR2A-Q1336E channels showed a reduced sensitivity to glibenclamide compared with the K_ATP_-WT complex (Figure 4D, 433 nM vs 4.3 nM, respectively). Additionally, the ATP sensitivity was right-shifted from 23.4 µM to 90 µM in Kir6.2-WT/SUR2A-Q1336E compared with control (Figure 4E). These data show that the Kir6.2-WT/SUR2A-Q1336E complex forms a functional channel, however some of the key modulatory characteristics have been lost.

### Kir6.2-D323K co-expressed with SUR2A-Q1336E, but not SUR2A-K1322D, partly restores the functional characteristics of the K_ATP_ complex

**Figure 5.**
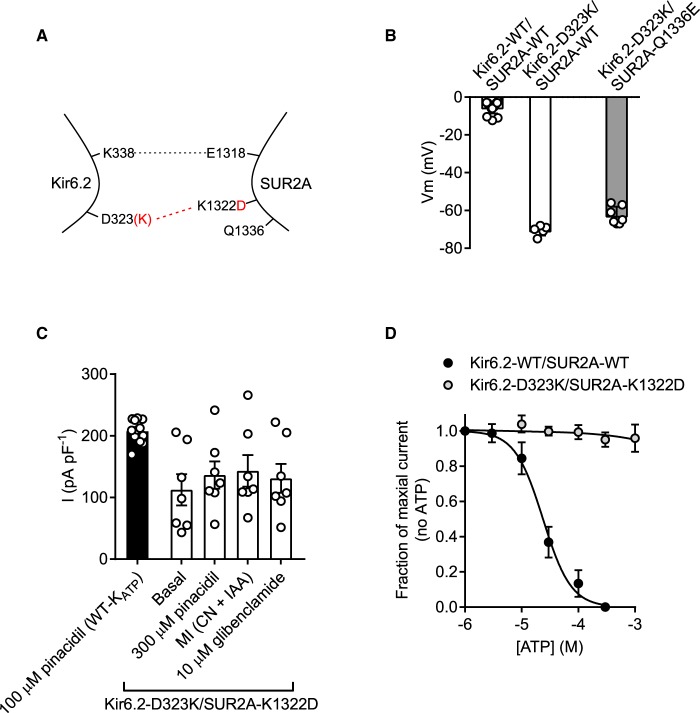
Co-expression of Kir6.2-D323K with SUR2A-K1322D to try to restore the salt bridge via a charge swop forms a channel that is constitutively active and does not respond to sulphonylureas or ATP. (**A**) Cartoon showing the Kir6.2-D323K and SUR2A-K1322D mutations potentially restoring the salt bridge interaction. (**B**) Mean membrane potential recording from HEK293 cells transiently transfected with Kir6.2-D323K/SUR2A-K1322D showing a hyperpolarised membrane potential comparable with the Kir6.2-D323K/SUR2A-WT (Vm data for Kir6.2-WT/SUR2A-WT and Kir6.2-D323K/SUR2A-WT from Figure 2 shown for comparison). (**C**) Mean whole-cell recording data, recorded at 0 mV, showing constitutive activity in basal conditions and no significant response to pinacidil, metabolic inhibition or glibenclamide (Repeated measured ANOVA, *n* = 7). WT-K_ATP_ activation with pinacidil shown for comparison. (**D**) ATP concentration inhibition data recorded in excised inside-out patches from WT-K_ATP_ and Kir6.2-D323K/SUR2A-K1322D showing that ATP had no inhibitory effect on the double mutant channel complex.

**Figure 6.**
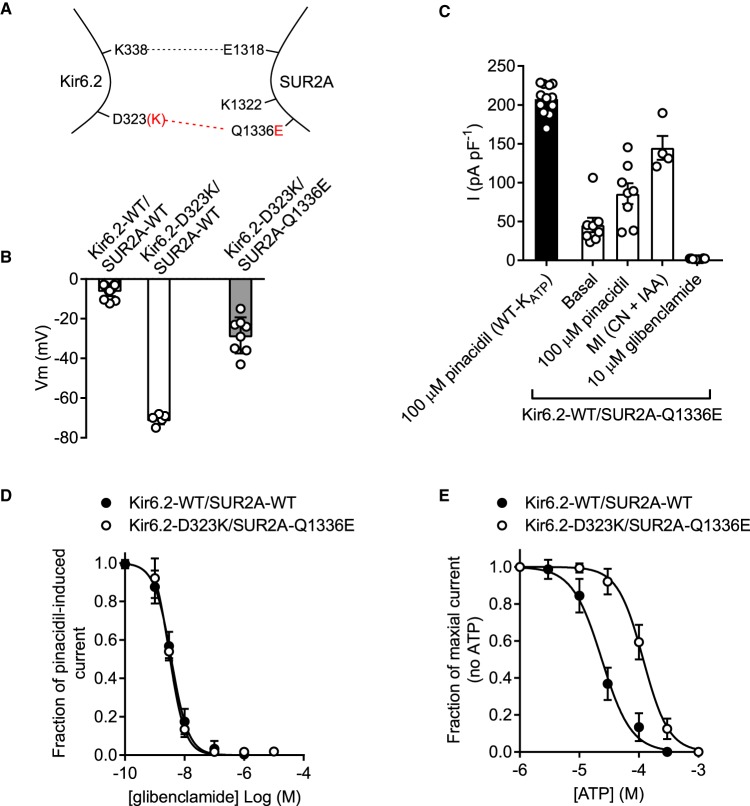
Co-expression of Kir6.2-D323K with SUR2A-Q1336E to try to restore the salt bridge via a charge swap forms a channel that shows some constitutive activity but is sensitive to sulphonylureas and ATP. (**A**) Cartoon showing the Kir6.2-D323K and SUR2A-Q1336E mutations potentially restoring the salt bridge interaction. (**B**) Mean membrane potential recording from HEK293 cells transiently transfected with Kir6.2-D323K/SUR2A-Q1336E showing a partially hyperpolarised membrane potential compared with WT-K_ATP_, however not as hyperpolarised as the Kir6.2-D323K/SUR2A-WT subunit combination (Vm data for Kir6.2-WT/SUR2A-WT and Kir6.2-D323K/SUR2A-WT from Figure 2 shown for comparison) (*n* = 8). (**C**) Mean whole-cell recording data, recorded at 0 mV, showing some constitutive activity in basal conditions that was enhanced by pinacidil and metabolic inhibition and fully reversed by 10 µM glibenclamide (Repeated measured ANOVA, *n* = 7). WT-K_ATP_ activation with pinacidil shown for comparison. (**D**) Glibenclamide concentration-inhibition data showing that the co-expression of Kir6.2-D323K/SUR2A-Q1336E fully restored glibenclamide sensitivity to the channel complex (IC_50_ of 3.9 nM and 3.7 nM for WT-K_ATP_ and Kir6.2-D323K/SUR2A-Q1336E, respectively, *t*-test, *P* = 1, *n* = 8). (**E**) ATP concentration inhibition data from WT-K_ATP_ and Kir6.2-D323K/SUR2A-Q1336E showing that the charge swap restored some ATP sensitivity, although this was right shifted from the WT-K_ATP_ (IC_50_ 23 µM and 123 µM, *n* = 6).

Co-immunoprecipitation data from our previous publication shows that the charge swap mutation in Kir6.2-D323K restores interaction with SUR2A-K1322D or SUR2A-Q1336E [14]. To confirm whether this charge swap could restore the normal regulation of channel gating by the SUR2A subunit to the constitutively active Kir6.2-D323K mutant, this mutant was co-expressed with either SUR2A-K1322D or SUR2A-Q1336E. The Kir6.2-D323K/SUR2A-K1322D combination showed constitutive activity and a hyperpolarised membrane potential with no effect of sulphonylurea drug or metabolic inhibition of the current (Figure 5B,C). Furthermore, this current was not ATP sensitive (Figure 5D). The Kir6.2-D323K/SUR2A-Q1336E subunit combination still showed some limited constitutive activity as evident by the slightly hyperpolarised membrane potential (Figure 6B), however also showed responsiveness to pinacidil, metabolic inhibition and to glibenclamide (Figure 6C). The Kir6.2-D323K/SUR2A-Q1336E showed a full restoration of glibenclamide sensitivity, being indistinguishable from K_ATP_-WT control (IC_50_ of 3.9 nM and 3.7 nM for K_ATP_-WT and Kir6.2-D323K/SUR2A-Q1336E, respectively) (Figure 6D). Unlike the Kir6.2-D323K mutant expressed with SUR2A or SUR2A-K1322D mutant, the Kir6.2-D323K/SUR2A-Q1336E was ATP sensitive, however it was significantly right shifted (IC_50_ 23 µM and 123 µM control and charge swap, respectively) (Figure 6E).

### Co-expression of Kir6.2-D323K with a double mutant SUR2A-K1322D/Q1336E does not restore functional parameters of the WT K_ATP_ complex

**Figure 7.**
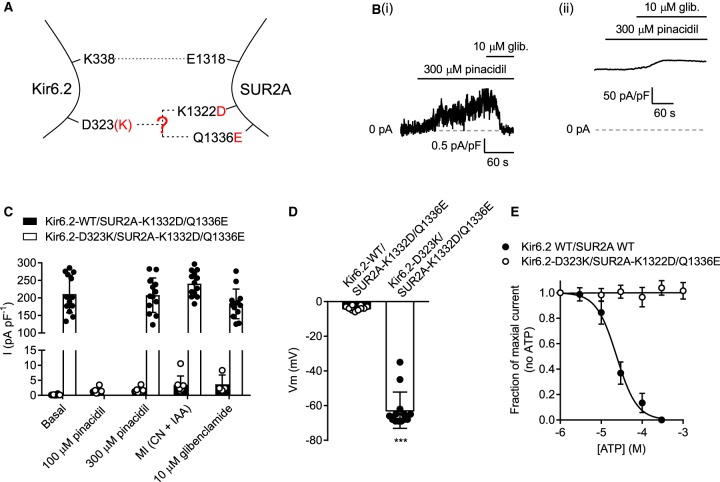
Co-expression of Kir6.2-D323K with a double mutant SUR2A-K1322D/Q1336E forms a channel that is constitutively active and insensitive to sulphonylurea drugs and ATP. (**A**) Cartoon showing the Kir6.2-D323K and SUR2A-K1322D/Q1336E mutations potentially restoring the salt bridge interaction. (**B**) Example whole-cell recordings at 0 mV from HEK293 cells transiently expressing Kir6.2-WT/SUR2A-K1322D/Q1336E showing a very small pinacidil and glibenclamide sensitive current (i) or Kir6.2-D323K/SUR2A-K1322D/Q1336E (ii) which showed a large, sulphonylurea insensitive and constitutively active current. (**C**) Mean whole-cell recording data, recorded at 0 mV, showing constitutive activity conditions that was unaffected by pinacidil, metabolic inhibition or glibenclamide (Repeated measures ANOVA, *n* = 13). (**D**) Mean membrane potential from both groups, showing that expression of the constitutively active Kir6.2-D323K/SUR2A-K1322D/Q1336E complex causes a significantly hyperpolarised membrane potential compared with expression of SUR2A-K1322D/Q1336E mutant with the Kir6.2-WT (*** *P* < 0.0001, *t*-test, *n* = 10). (**E**) ATP concentration inhibition data from WT-K_ATP_ and Kir6.2-D323K/SUR2A-K1322D/Q1336E showing that the mutated complex is not ATP-sensitive (*n* = 4).

It was hypothesised that as both SUR2A-K1322D and -Q1336E mutations co-immunoprecipitated with Kir6.2-D323K in our previous study [14], that perhaps Kir6.2-D323 interacted with both residues on the SUR2A subunit in the fully formed channel complex. To investigate this, a double SUR2A mutant was expressed in HEK293 cells with the Kir6.2-D323K mutant. Expression of Kir6.2-WT with the SUR2A-K1322D/Q1336E double mutant yielded a current that, although pinacidil and glibenclamide sensitive, was too small to record meaningful data from (Figure 7B,C). Co-expression of the Kir6.2-D323K/SUR2A-K1322D/Q1336E complex yielded a channel that was constitutively active, sulphonylurea drug insensitive and ATP insensitive (Figure 7B–E).

### Co-expression of the Kir6.2-D323K equivalent mutation in Kir6.1 (E332K) with SUR2A-K1322D, but not Q1336E, restores glibenclamide sensitivity to a constitutively active Kir6.1

**Figure 8.**
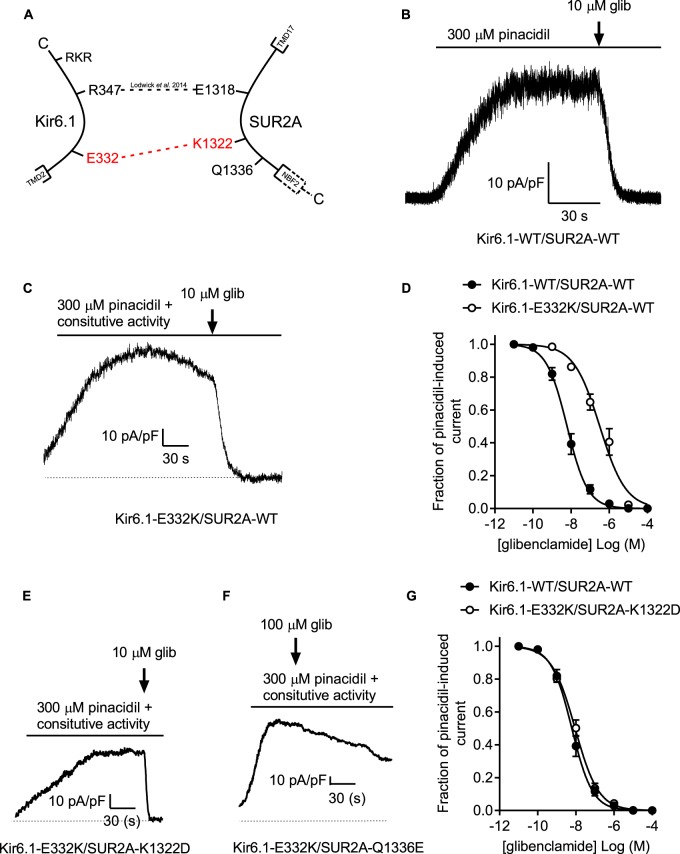
A salt bridge between the equivalent Kir6.1 residue (E332) and SUR2A exists with K1322 rather than Q1336. (**A**) cartoon showing the putative interaction with Kir6.1-E332 and SUR2A-K1322. (**B**) Example trace of whole-cell Kir6.1/SUR2A current activated by pinacidil. (**C**) Whole-cell trace showing constitutively active Kir6.1 current, enhanced with pinacidil and inhibited with 10 µM glibenclamide. (**D**) Concentration inhibition curve for Kir6.1-WT/SUR2A-WT and Kir6.1-E332K/SUR2A-WT showing a rightward shift in with the mutated Kir6.1 pore (*n* = >6 per data point, IC_50_ values of 6.13 ± 1.2 to 318 ± 11 nM in WT and mutants, respectively). Example traces showing Kir6.1-E332K co-expressed with the charge swap SUR2A mutants SUR2A-K1322D (**E**), showing constitutive activity and responsiveness to glibenclamide, and SUR2A-Q1336E (**F**) showing constitutive activity that was unresponsive to 100 µM glibenclamide. (**G**) concentration response curve for glibenclamide showing no difference between Kir6.1-WT/SUR2A-WT and Kir6.1-E332K/SUR2A-K1322D channels, (*n* = >6 per data point, IC_50_ values of 6.1 ± 1.2 to 9.2 ± 3.1 nM in WT and mutant, respectively.

Kir6.1 is a markedly different pore forming subunit to Kir6.2 in that it shows little ATP-dependence, but that is highly regulated by kinases and ADP. These modulators are suggested to act via the SUR subunit and so a similar transfer of information must occur between these conserved interacting residues. This has been previously reported for a residue in Kir6.1-E347 where a mutant Kir6.1-E347R assembled with SUR2A-D1318E and restored sulphonylurea drug sensitivity [14]. In this current study, we identified that the Kir6.1-E332K mutant expressed with SUR2A-WT also yielded a constitutively active channel, like its Kir6.2-D323K counterpart, however showed right-shifted glibenclamide sensitivity rather than completely abolishing it Figure 8B–D). Co-expression of Kir6.1-E332K with SUR2A-K1322D restored glibenclamide sensitivity to that indistinguishable from Kir6.1-WT/SUR2A-WT, however co-expression of Kir6.1-E332K/SUR2A-Q1336E did not restore the glibenclamide sensitivity (Figure 8E–G). These data suggest that Kir6.1-E332K interacts with SUR2A-K1332 whereas Kir6.2-D323 interacts with SUR2A-Q1336.

### Mutation of Kir6.2-D323 does not cause the pore-forming subunit to become constitutively active

**Figure 9.**
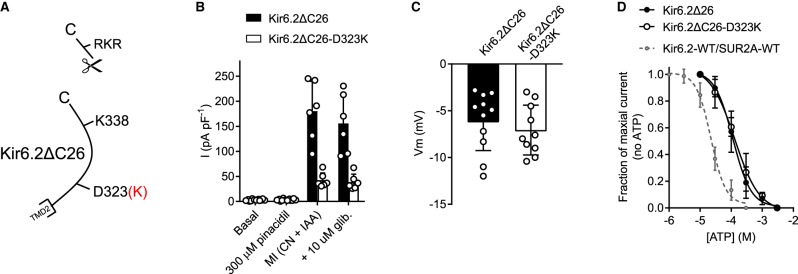
Truncation of the Kir6.2-D323K mutation to remove the SR-retention sequence yields a channel that is not constitutively active and ATP sensitive. (**A**) Cartoon showing the truncation of Kir6.2. (**B**) Mean whole-cell data recorded at 0 mV from Kir6.2ΔC26 and the Kir6.2ΔC26-D323K mutant showing neither channel is constitutively active, neither responds to sulphonylurea drugs, but both were activated by ATP depletion with metabolic inhibition (cyanide and iodoacetic acid) (*n* = ≥6 for each group). (**C**) Mean membrane potential recordings showing no difference in resting membrane potential between the WT and mutated truncation (*n* = ≥10 for each group). (**D**) The ATP sensitivity of the Kir6.2ΔC26 truncated channel is shifted 10-fold compared with Kir6.2/SUR2A (IC_50_ 123 µM compared with 23 µM in Kir6.2ΔC26 compared with Kir6.2/SUR2A, respectively) however the Kir6.2ΔC26-D323K mutant showed no difference in its ATP sensitivity compared with the Kir6.2ΔC26 mutant expressed alone.

To determine whether mutation of Kir6.2-D323 is the cause of the switch from ATP-sensitive to constitutively active, a modified Kir6.2ΔC26 truncation mutant was used. The deletion of the C-terminal 26 amino acid removes the RKR-endoplasmic reticulum retention sequence that is usually masked by co-assembly with the SUR accessory subunit. This Kir6.2ΔC26 truncation mutant can express without the SUR, and retains ATP sensitivity [12], albeit right shifted 10-fold as the presence of the SUR enhances the ATP sensitivity. Kir6.2ΔC26 truncation, or Kir6.2ΔC26-D323K, was expressed in HEK293 cells. Both were expressed, and both showed activation in response to ATP depletion with metabolic inhibition (CN and IAA). Both Kir6.2ΔC26 and Kir6.2ΔC26-D323K were insensitive to pinacidil or glibenclamide, as would be expected with no SUR co-expression, but neither channel showed constitutive activity in whole-cell recording (Figure 9B,C). Furthermore, both channels were ATP-sensitive with an IC_50_ of ∼123 µM (Figure 9D), comparable with previous literature for the Kir6.2 truncation, and right shifted ∼10-fold from the Kir6.2/SUR2A complex [12]. These findings suggest that the Kir6.2-D323K mutation does not itself cause the constitutive activity in the channel.

**Figure 10.**
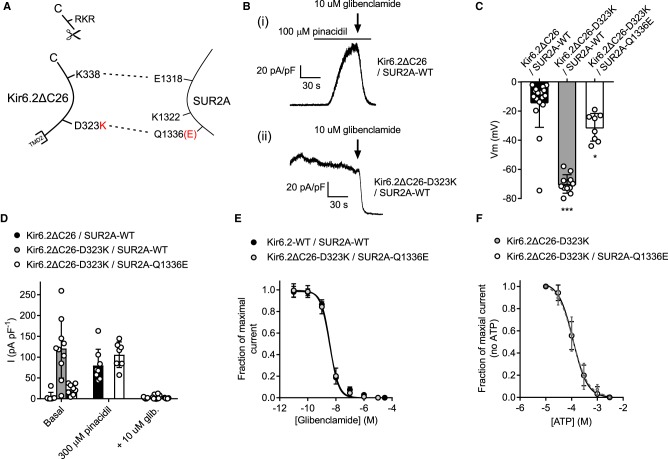
Kir6.2ΔC26-D323K co-expression with SUR2A-Q1336E restores sulphonylurea sensitivity however the channel remains partially constitutively active. (**A**) Cartoon showing the truncation of Kir6.2 and the putative interaction with SUR2A between residues D323 of Kir6.2 and Q1336 of SUR2A. (**B**) Example traces from (i) Kir6.2ΔC26 and (ii) Kir6.2ΔC26-D323K co-expressed with SUR2A-WT. Kir6.2ΔC26-D323K/SUR2A-WT co-expression forms a constitutively active current, whereas the Kir6.2ΔC26/SUR2A-WT combination requires pinacidil to activate the current. Both combinations are inhibited by glibenclamide. (**C**) Mean membrane potential data from Kir6.2ΔC26 /SUR2A-WT, Kir6.2ΔC26-D323K/SUR2A-WT and Kir6.2ΔC26-D323K/SUR2A-Q1336E, suggesting some constitutive activity with a slightly hyperpolarised membrane potential (*** *P* < 0.0001, * *P* < 0.05, one-way ANOVA with Holm-Sidak's post-test, *n* = >8). (**D**) Mean current recording from Kir6.2ΔC26/SUR2A-WT, Kir6.2ΔC26-D323K/SUR2A-WT and Kir6.2ΔC26-D323K/SUR2A-Q1336E combinations showing some constitutive activity in the charge swap pairing (*n* = >8). Kir6.2ΔC26-D323K/SUR2A-WT was constitutively active and not further enhanced by pinacidil. (**E**) Concentration-response data for glibenclamide showing that co-expression of Kir6.2ΔC26-D323K/SUR2A-Q1336E charge swap mutations had identical inhibition profile to Kir6.2-WT/SUR2A-WT co-expression (*n* = >6 for each data point, IC_50_ of 3.8 ± 1.1 and 3.6 ± 0.6 in WT-truncation mutant and double mutant, respectively). (**F**) ATP sensitivity of the Kir6.2ΔC26-D323K/SUR2A-Q1336E charge swap mutants showing identical inhibition by ATP to the Kir6.2ΔC26 truncation mutation (*n* = 6 for each data set, IC_50_ of 113 ± 5 and 114 ± 5 µM for WT-truncation and double mutant, respectively), but not Kir6.2-WT/SUR2A-WT (23.8 ± 1.2 µM, shown in Figure 2).

Co-expression of Kir6.2ΔC26-D323K with SUR2A-WT results in a channel that is constitutively active, as seen in the full length D323K mutant in Figure 2, however that was sensitive to glibenclamide (Figure 10). Given the instability of the current, and the lack of sensitivity to pinacidil to try to stabilise the opening, it was not possible to record a concentration response curve.

Data to this point has suggested that the SUR2A-Q1336 residue gives the closest to WT phenotype when co-expressed with the Kir6.2-D323K mutant. With this Kir6.2 mutant, even when co-expressed with the SUR2A-Q1336E, there was still constitutive activity. Co-expression of Kir6.2ΔC26-D323K/SUR2A-Q1336E resulted in channels that showed constitutive activity but retained pinacidil, metabolic inhibition and glibenclamide sensitivity (Figure 10B–D). The Kir6.2ΔC26-D323K/SUR2A-Q1336E complex also retained ATP sensitivity (Figure 10E), however it remained 10-fold right shifted suggesting that, despite the presence of the SUR accessory subunit, and this mutation did not restore the full ATP-sensitivity seen with the K_ATP_-WT.

## Discussion

**Figure 11.**
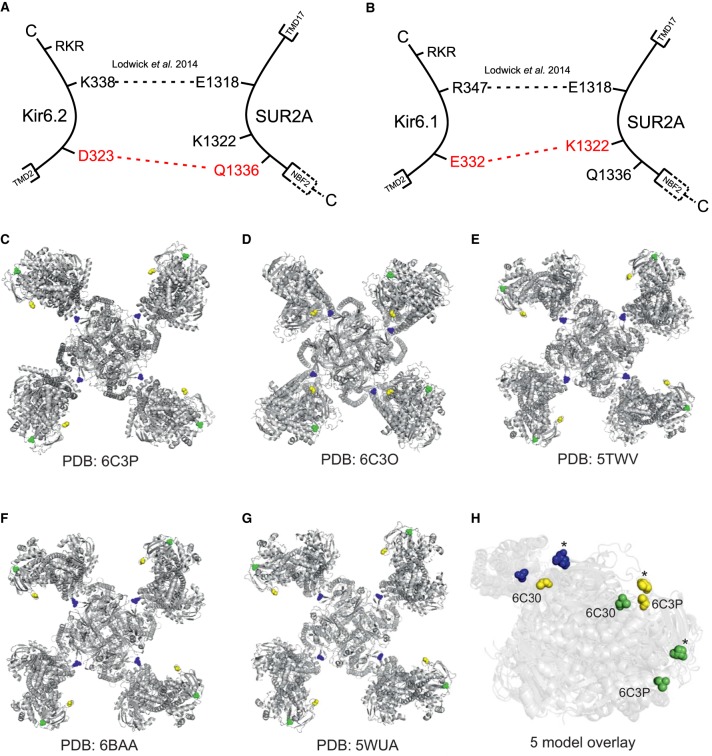
Cartoon representation of the interactions between Kir6.2 and Kir6.1 and the SUR2A accessory subunit and the position of the identified residues. Cartoon representation of the putative interaction between Kir6.2-D323 and SUR2A-Q1336 (**A**) and Kir6.1-E322 and SUR2A-K1322 (**B**). Images **C**–**G** show the location of Kir6.2-D323 (blue), SUR-K1322 (yellow) and SUR-Q1336 (green) on published K_ATP_ complex structures showing a core tetramer of Kir6.2 surrounded by four SUR1 Subunits. Structures from Protein Data Bank, (**C**) PDB code:6C3P [32], (**D**) PDB code:6C3O [32], (**E**) PDB code:5TWV [31], (**F**) PDB code:6BAA [30], (**G**) PDB code:5WUA [29]. (**H**) Five model overlay of **C**–**G** showing a single SUR2A and Kir6.2 to demonstrate movement of key residues within published structures. * indicates residues that do not move in models 5TWV, 6BAA and 5WUA.

The data from this study suggests that there is a physical interaction between residues D323 on Kir6.2 and Q1336 of SUR2A (Figure 11A). This interaction allows the transfer of information between the SUR and Kir6 subunits with respect to channel gating in the presence of sulphonylurea drugs, such as glibenclamide. Furthermore, we show that a conserved charge at the analogous position in Kir6.1 (E332K) interacts with SUR2A, however at an alternative residue than Kir6.2, SUR2A-K1322 (Figure 11B). These data presented here functionally characterise a further electrostatic interaction between the C-terminal domain of Kir6.2 and the nucleotide binding domain 2 in the proximal C-terminal region of the SUR2A subunit. As with our previously identified electrostatic interaction (Kir6.2-K338/SUR2A-E1318) [14], disruption of the pairing between Kir6.2-D323/SUR2A-Q1336 alters the regulation of the pore by ATP, glibenclamide and pinacidil, with the near complete restoration of function on repair of the electrostatic interaction by opposite charge swap mutations. This highlights the importance of these regions of the proteins for the interaction between the Kir6 and SUR subunits. The main functional data from the Figures 1 to 10 are summarised in Table 1.

**Table 1:** Summary of the main functional data recorded for Kir6.2 and SUR2A mutants in the study

Kir6.2	SUR2A	Constitutive activity?	Vm (mV)	Glibenclamide IC_50_ (nM)	ATP IC_50_ (µM)
WT	**WT**		−4 ± 1	4 ± 1	24 ± 1
D323K	**WT**	✓✓	−71 ± 1	>100 µM	>10 mM
WT	**K1322D**		−4 ± 1	†	†
D323K	**K1322D**	✓✓	−63 ± 2	>100 µM	>10 mM
WT	**Q1336E**		−11 ± 1	433 ± 6	90 ± 7
D323K	**Q1336E**	✓	−29 ± 3	4 ± 1	123 ± 2
WT	**Q1336 + K1322D**		−4 ± 1	†	†
D323K	**Q1336 + K1322D**	✓✓	−63 ± 3	>100 µM	>10 mM
ΔC26	**–**		−6 ± 3	–	120 ± 4
ΔC26-D323K	**–**		−7 ± 3	–	113 ± 5
ΔC26-D323K	**WT**	✓✓	−70 ± 2	–	–
ΔC26-D323K	**Q1336E**	✓	−31 ± 3	4 ± 2	123 ± 6
Kir6.1-WT	**WT**		–	6 ± 1	–
Kir6.1-E322K	**WT**	✓✓	–	318 ± 11	–
Kir6.1-E322K	**K1322D**	✓	–	9 ± 3	–

✓✓, Constitutive activity causing significant, often irreversible, channel activity.

✓, Some constitutive activity that could be enhanced with pinacidil and fully reversed with glibenclamide.

†, Current too small to measure accurately.

Kir6.1/SUR2A data has also been included for completeness.

This newly identified region of interaction between the C-terminal regions of Kir6 and SUR2 subunits is distinct from reported interactions, with alternative regions previously identified in other studies. Interaction between TMD0 and the M2 TMD of Kir6 subunits has previously been deemed important for the assembly of the channel [33]. Additionally, isoform-specific regulation by nucleotides of channel opening has also been linked to the L0 linking sequence between TMD0 and TMD1 in SUR subunits [33–35]. Here we report a second electrostatic interaction between a single residue (D323) on Kir6.2 at the C-terminal region and a corresponding charge partner (Q1336) on the C-terminal region of SUR2A.

Our earlier studies [14,15,36] focussed on this C-terminal interacting region as a point of allosteric information transfer between the heterologous subunits. There were changes in the functional parameters of the channel when the Kir6.2-K338/SUR2A-E1318 electrostatic interaction was broken, which were restored with a direct charge swap. Our data has been further strengthened with the recent Cryo-electron microscopy structures of Kir6.2 co-expressed with SUR1 [37]. In this study, the authors suggest that our identified Kir6.2-K338 residue comes into close proximity with the proposed charge partner, the equivalent of SUR2A-E1318, in one of the three conformations identified [37].

In this present study, it was hypothesised that Kir6.2-D323K/SUR2A-Q1336E charge swap would restore the function of the channel complex as the reciprocal charge swap for our previous pairing had shown [14]. Single charge mutations, such as Kir6.2-D323K co-expressed with SUR2A-WT, had the surprising effect of causing a constitutive activity in the normally electrically silent channel complex. The apparent lack of inhibition by ATP for this Kir6.2-D323K with the SUR2A-WT suggests that there has been either a disruption of the ATP-binding site, or a disruption of the interaction between Kir6.2 and SUR2A that modulates normal gating. Evidence for a disruption of gating rather than an alteration of the Kir6.2 ATP binding site comes from the Kir6.2ΔC26-D323K mutant where expression of this truncated and mutant pore-forming subunit yields a current that is not constitutively active and is ATP sensitive. Furthermore, co-expression with the charge swap SUR2A-Q1336E mutant fully restores channel function with the exception of the ATP-sensitivity, which remains at ∼120 µM consistent with the truncation of Kir6.2 expressed alone [12]. Perhaps somewhat unexpectedly, the Kir6.2ΔC26-D323K mutant when expressed with SUR2A-WT did form a channel showing substantial constitutive activity that was insensitive to pinacidil, however was fully inhibited by 10 µM glibenclamide. These findings suggest that this single point mutation triggers something deleterious to the formation of a normally functioning channel complex with the full length Kir6.2-D323K mutant compared with the truncated mutant, Kir6.2ΔC26-D323K, co-expressed with the SUR2A-WT. Perhaps the interaction with the RKR motif of the full length Kir6.2 protein has a constraining effect on the correct interaction between Kir6.2-D323 and SUR2A-Q1336.

Constitutive activity of Kir6.1-E332K, the analogous residue to Kir6.2-D323, was also seen when co-expressed with SUR2A-WT. In this case, the co-expression of Kir6.1-E332K with the SUR2A-K1322D, but not the SUR2A-Q1336E, fully restored glibenclamide sensitivity to the complex. These findings suggest that Kir6.1 and Kir6.2 interact at alternative SUR2A residues, K1332 and Q1336, respectively. The similarities in pharmacological response suggest that there is a common zone of interaction between SUR2A-E1318 and Q1336 and the proximal C terminus of the Kir6 pore forming subunits despite the regulatory differences in these proteins. It has been suggested that the relative insensitivity of Kir6.1 to ATP, that largely fail to open spontaneously in the absence of ATP, results from an enhanced response to stimulatory ATP binding to SUR rather than a reduced sensitivity to the inhibitory binding of ATP to the Kir6.1 subunit [24].

Dupuis et al. [38] used a similar approach to ours to identify three residues in SUR2A which, when changed to the equivalent residues in the non-interacting maltose binding protein (MBP1), were sufficient to attenuate the activation of the channel complex by ADP and potassium channel openers. Expanding this, Prinicpalli et al. demonstrated further evidence that the region identified by our previous studies in the SUR [14,15], included additional residues in SUR1 (Q1342, I1347, and L1350) that confer allosteric regulation on channel gating [39]. These studies add to the understanding of the complexity of the mechanisms behind allosteric information transfer between SUR and Kir6 subunits.

**Figure 12.**
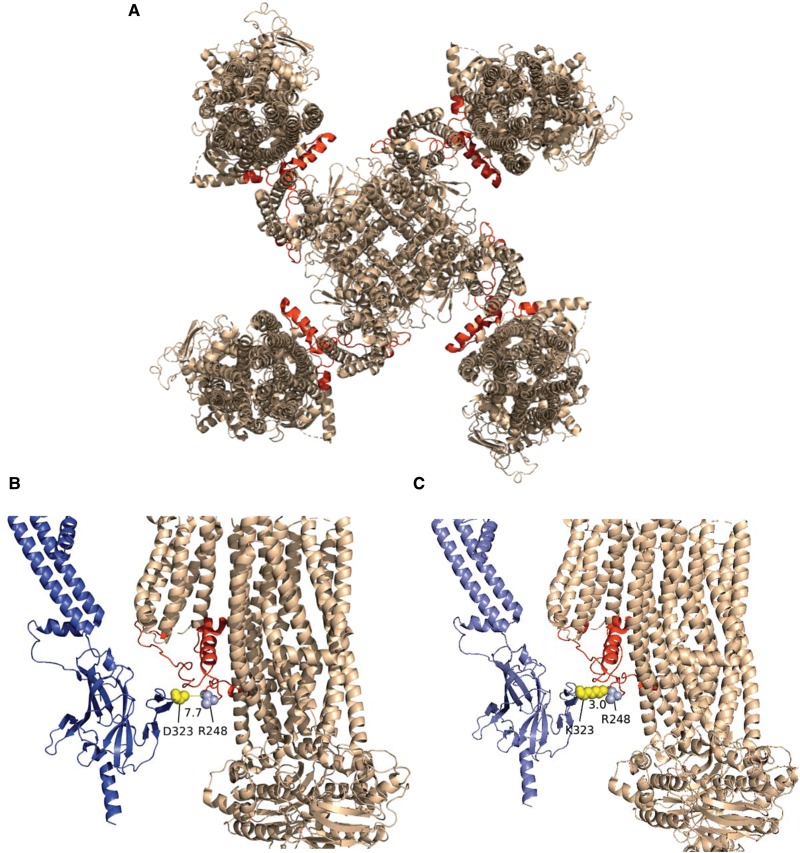
Kir6.2-D323 is in close proximity to a highly-conserved Lasso region within SUR subunits (residues 193–261) known to be involved in regulation of gating. (**A**) Ribbon representation of SUR1-Kir6.2 channel complex in propeller conformation (PDB code: 6C3P [32]) modelled using Pymol software. Red colour indicates the Lasso region (residues 193–261). (**B**) Expansion of SUR1-Kir6.2 channel complex in ribbon representation, illustrating the distance between D323 and R248 on the lasso region. In this structure; Kir6.2 is in purple, SUR1 is in cream and the lasso region is shown in red. (**C**) Expansion of SUR1-Kir6.2 channel complex in ribbon representation, illustrating the effect of D323K mutation on the distance between residue 323 and R248 (the lasso region). D323K mutation brings K323 within 3 Å of R248 on the Lasso region. As with part B, in this structure Kir6.2 is in purple, SUR1 is in cream and the lasso region is shown in red.

With the recent cryo-EM structure of the Kir6.2/SUR1, we have new insight into the folding and alignment of the subunits within the K_ATP_ channel complex [37]. In the three main structural patterns published, it is unlikely that our identified residues of interaction could form any permanent salt bridge interaction. The D323 residue is located on an outside face of the cytoplasmic domain that brings it within close proximity of a highly-conserved region within SUR subunit (the ‘lasso region' residues 193–261) [32,37], whilst the SUR2A-Q1336 equivalent in the SUR1 model is located on a region located in the lipid environment externally facing the channel complex (Figure 11C–E, identified as green). The SUR subunit in particular shows a significant degree of flexibility between the five published structures shown in Figure 11C–G, with an overlay of the five models shown in Figure 11H for comparison. This suggests that the interactions that we have described in this manuscript may be important for the correct folding *between* orientations rather than a key functional structure. Interestingly, the lasso region (193–261), which is in close proximity to D323 (Figure 12A), has been previously shown to be important in gain-of-function mutations in some diabetic states [32,40]. Through these mutations, the open-state of the K_ATP_ channel is stabilised and the ATP-inhibition of the channel is overcome, both of which lead to over-activity of the channel [32]. Examples of residues involved are R248 deletion (located on the Lasso extension) which led to immature SUR1 subunit formation and ultimately to hyperinsulinism of infancy and Y330 (located on the ATP binding pocket and therefore adjacent to the lasso extension) which led to permanent neonatal diabetes [40].

Given that our electrophysiology and previous co-immunoprecipitation data suggest some form of interaction is possible between these residues, we suggest that the degree of flexibility possible within the SUR2A subunit could allow the formation of a temporary interaction that perhaps stabilises a transition state between the activated and inactive conformations. Alternatively, this interaction may occur between adjacent Kir6.2-SUR heteroctoamers or an additional protein, as yet unidentified, may interact with the K_ATP_ complex and these residues form part of this interaction. It is also possible that this interaction represents conformations of Kir6.2-SUR2A that have yet to be visualised using cryo-EM as the current models are based on SUR1.

Interestingly, using the current cryo-EM structures, modelling of the D323K mutation changes the distance between the Kir6.2-D323 and the SUR-R248 residues from 7.7 Å to 3 Å (Figure 12B,C). We suggest that this may cause the change from a physiologically silent to constitutively active channel in the D323K mutant. Whilst D323 is not located immediately at the ATP-binding pocket on the Kir6.2 subunit, it is in close proximity to the lasso extension which plays important role in regulating the function and gating of K_ATP_ channels. Disruption of the lasso extension has been shown to cause significant conformational changes in K_ATP_ channel (propeller and quatrefoil forms) [32], and point mutations along the lasso extension as well as to residues adjacent to it have been observed to associate with gain-of-function of K_ATP_ channel [40,41]. Therefore, we suggest that the Kir6.2-D323K mutation elicits its effect via its disruptive influence on the lasso extension, so modulating ATP sensitivity, but perhaps fails to allow the full appropriate interaction with the SUR2A-Q1336E ‘equivalent’ charge swap during the transition between states.

In summary, an interface between heterologous K_ATP_ subunits was identified containing an electrostatic interaction between Kir6.2-D323 and SUR-Q1336 residues, and the equivalent residue on Kir6.1, Kir6.1-E332, and alternate SUR2A residue K1332. This interaction, together with our previously identified interaction between Kir6.2-K338 and SUR2A-E1318, are important sites of information transfer between heterologous K_ATP_ subunits determining sensitivity to allosteric regulators.

## Abbreviations

HEK: human embryonic kidney
MBP: maltose binding protein
NDP: nucleotide diphosphate
SUR: sulphonylurea receptor
WT: wild-type

## Open Access

Open access for this article was enabled by the participation of University of Liverpool in an all-inclusive *Read & Publish* pilot with Portland Press and the Biochemical Society under a transformative agreement with JISC.
